# Impact of quantitative safety targets on road fatality reduction: an empirical support toward governance plan

**DOI:** 10.3389/fpubh.2023.1271328

**Published:** 2023-11-07

**Authors:** Haizhou Cui, Yuliang Guo, Yuchen Li, Jianwei Zhang, Yida Wang, Lin Yang, Jiayi Hu, Hak Kei Wong, Yuxuan Bai, Yang Ma, Faan Chen

**Affiliations:** ^1^School of Economics, University of Sydney, Camperdown, NSW, Australia; ^2^Division of Arts and Humanities, Las Positas College, Livermore, CA, United States; ^3^School of Health Sciences, University of Manchester, Manchester, United Kingdom; ^4^College of Arts and Sciences, Georgia State University, Atlanta, GA, United States; ^5^Harris School of Public Policy, University of Chicago, Chicago, IL, United States; ^6^Krieger School of Arts and Sciences, Johns Hopkins University, Baltimore, MD, United States; ^7^Department of Mathematics, Columbia University, New York, NY, United States; ^8^Faculty of Life Sciences, University College London, London, United Kingdom; ^9^Department of Biomedical Engineering, University of California at Irvine, Irvine, CA, United States; ^10^School of Culture, Education, and Human Development, New York University, New York, NY, United States; ^11^School of Engineering and Applied Sciences, Harvard University, Cambridge, MA, United States

**Keywords:** public health, road fatality, quantitative target, target setting, panel data, OECD

## Abstract

**Introduction:**

The role of quantitative target setting has become an important topic in debates on the improvement of road safety performance. Specifically, there are questions regarding the relationship between quantitative safety targets and their actual effects. Although previous studies have provided important insights into this subject, their empirical findings have largely been equivocal, and research on this topic remains inadequate.

**Methods:**

Based on panel data representing 20 years of observations from 34 OECD member states, we employed nonlinear and linear panel models to investigate whether and how the attributes of quantitative road safety targets (i.e., target ambition and duration) influence their success (i.e., target completion status and rate).

**Results:**

The results indicate that a quantitative target with a higher level of ambition is associated with a lower likelihood and rate of completion, whereas there is no support for a connection between target duration and final completion rate. This suggests that an excessively ambitious target does not necessarily result in better road safety performance and is detrimental to achieving expected fatality reductions.

**Conclusion:**

From an empirical perspective, this study revealed a potential interaction effect between quantitative road safety targets and practical fatality reduction performance, providing government officials and policymakers with essential references for future practices on target setting and governance planning in regard to public health.

## 1. Introduction

The number of road traffic deaths worldwide remains unacceptably high, with 1.35 million people dying each year (1). Although many countries are taking action, road traffic deaths remain the dominant safety hazard in the daily lives of citizens (2) and as the eighth leading cause of death globally. Organizations and governments in different countries have regularly adopted quantitative targets to reduce road fatalities. For example, the application of quantitative targets as guidelines for road safety construction by EU countries is an archetypal success in modern transportation development (3). In particular, the Vision Zero road safety policy also revolutionarily envisions zero deaths as the ultimate road safety goal, which can be regarded as a new mile-stone of safety policy regulation with prospects (4, 5).

However, setting ambitious but achievable quantitative targets is challenging. The whole target-setting process is fraught with challenges, particularly in relation to the determination of the core characteristics of targets (e.g., target ambition and target duration). Policymakers should have explicit ideas regarding ambitions and timeframes in the target-setting process. Previous empirical studies have provided valuable insights into these factors and established a solid foundation for target setting (6–11), but their findings are equivocal and widely debated in academia with two types of antithetical opinions. Some studies have found that the key characteristics of targets, such as the level of targeted fatality reduction and durability of target execution, have a significant positive relationship with the likelihood of their achievement (7, 12, 13), whereas others claim that it is a negative relationship (8, 14, 15). These studies were mostly conducted within individual countries, from which the findings may not be applicable and generalizable to other parts of the world. In practice, no guidelines or normative theories exist regarding the choice of attributes.

The essence and importance of the study on the impact of quantitative safety targets on realized road fatality reduction toward governance plan can be extended and sublimated to a higher conceptual level. The process of governance planning and public policy development is commonly defined as a purposive course of action taken by one or more individuals or groups to address a particular problem or concern (16). Positioning the process of public policy should be synthetical, and the concentration should encompass the whole steps, including adoption, implementation, following-up, and evaluation, which form a typical political decision-making framework (17, 18). Adoption provides a starting point for implementation while also offering a reference point for future evaluations (19). Therefore, the effectiveness of a road safety policy could be comprehensively analyzed only when the all-inclusive step of the framework is considered. A particular success of a comprehensive analysis can be referred to an examination of Sweden’s Vision Zero road safety policy, which contributes to demonstrating the overall important aspects of road safety policy (5). Wherein, on the basis of which general principles the road safety goal is to be achieved motivates our in-depth research on the effect of implementing quantitative safety targets on actual reductions in road fatalities. Setting quantitative goals only stops at the adoption level (too narrow in terms of defining a road safety policy), but it is more important to understand and evaluate its policy implications. Quantitative target setting epitomizes the ambition and duration of a government to control road safety risks. Governmental officials need to understand whether the government’s ambition and duration can be the general principles the road safety goal is to be achieved and at what level they should be when making governance plans and transport policies. Therefore, it is crucial to clarify these relationships using a reliable dataset with a large sample and long timespan to support future target setting endeavors. However, research in this area is still insufficient.

With the goal of filling this gap, we employed panel models (i.e., linear and nonlinear) to derive empirical indications of any potential relationship between the ambition of target setting and the final completion status using panel data representing 20 years of observations from 34 OECD countries. With such a large sample and longitudinal observations based on rigorous panel data models, individual heterogeneity can be eliminated such that the general relationships between quantitative target attributes and target achievement can be clarified without the influence of country individuality. Our findings help government officials, policymakers, and practitioners to systematically understand the underlying rationale behind road safety risk management and provide them with additional evidence and reliable references for future quantitative target settings and political decision-making.

The remainder of this paper is organized as follows. Section 2 reviews previous studies in this area. Section 3 describes the dataset used in this study and the associated resources. Section 4 details the methodology employed in this study, which is followed by empirical results and discussion in Section 5. Section 6 summarizes our main findings and describes potential directions for future research.

## 2. Literature review

The establishment of quantitative safety targets for the reduction of road fatalities is a practice that has been widely adopted by administrative bodies and countries over the past two decades. However, few relevant studies have explored how effectively a quantitative safety target can contribute to better road safety performance or how to identify the attributes of targets that are achievable.

### 2.1. Quantitative targets and road safety performance

The effectiveness of quantitative safety target setting is hotly debated in the context of providing supportive guidance for authorities to take action [e.g., (7, 20–23)]. Given that setting quantitative targets is an essential element of policy-related road safety strategies, road safety targets must be quantifiable to determine whether they have been accomplished or the degree to which the outcomes fall short of goals (24, 25).

In practice, multiple safety performance metrics linked to roadway infrastructure, automotive technology, and user behavior can be produced. Tolón-Becerra and Lastra-Bravo (26) proposed a straightforward approach to allocating the collective effort required to achieve the EU’s quantitative target of reducing fatalities. A comprehensive safety performance metric involving setting a series of appropriate quantitative target attributes for road safety control can be utilized as a management instrument for defining suitable road safety targets while designing effective road safety strategies and countermeasures (27). Therefore, an improved understanding of setting optimal quantitative targets and their correlation with current road safety management systems in terms of the anticipated impact of time series trends on road safety should significantly reduce road fatalities and improve safety performance.

To evaluate the relationship between quantitative target setting and road safety performance, various analytical frameworks have been applied. In-depth analytical approaches have been adopted to interpret the association between road safety progress made by authorities and the measurements they collect, including assessments of target setting appropriateness [e.g., (28, 29)]. Furthermore, realistic and measurable road safety targets can be established by referencing both legislative and engineering interventions and their implementations. This can be accomplished while accounting for any time series trends in road safety levels (30), particularly the effects of quantitative target setting on the time series trends of road fatalities [e.g., (9, 13)]. Analytical approaches using statistical inference are widely used to construct empirical relationships between quantitative target setting and the realized outcomes of road safety performance, given that the adopted methodologies can objectively identify implications among variables. Such methods include cluster analysis and autoregression (31). Furthermore, regional studies referring to individual countries and areas have also been conducted, and regional uniqueness in terms of socio-economic development conditions has been considered [e.g., (12, 14, 15, 22)].

### 2.2. Attributes of quantitative safety targets and their achievement

Is a country with more ambitious safety targets more successful at achieving its goals? Arguments regarding this point present two types of antithetical opinions. Some studies claim that more ambitious quantitative targets for traffic fatality reduction are more likely to result in success, whereas others indicate that overly ambitious target setting may have counterproductive effects.

A quantitative target is the expression of political will by an organization or country to improve road safety. In general, governments with more ambitious targets are more inclined to take action to achieve better performance in terms of road injury and fatality reduction (7), so they are more likely to support proposed policy and legislative changes, and allocate sufficient resources to safety programs. This indicates that effective road safety risk control requires a government with strong intentions to take measures and that road safety conditions with a motivated government are better than those with an indifferent government. Furthermore, the overall reduction in road fatalities can be conspicuous because setting an ambitious target can raise social concern and increase the attention devoted to transportation safety issues, inducing more willingness in society to allot resources to road safety programs and incentivizing local governments (13). This concept is supported by a study indicating that to reduce cyclist injuries, a government must define a strong target to direct society to take action (32). In other words, a greater-than-average improvement in road safety performance is likely to be achieved in countries and areas with high-level and long-term ambitious targets (33).

Some studies have found that if targets are defined too ambitiously to be achieved by the current road safety strategy, then they may lose the motivating effects that challenging, but achievable targets often carry (11, 34) because public managers will feel reluctant to take measures if the targets are too ambitious. The successful implementation of road safety strategies often requires realistic target setting (35, 36). Strict time-bounded targets are generally difficult to achieve and their theoretical feasibility is poor (14). In contrast, less ambition in setting fatality reduction goals will generate a greater likelihood of achievement (8).

In general, there should be a balance between ambition and target-achieving capabilities. A lack of confidence will lead to limited road safety performance, but overconfidence may impair the validity of execution (14). An excessively ambitious target may cause demotivation because desire without high-level execution ability will lead to incomplete implementation (8, 34). In summary, better performance of road safety development and traffic fatality control is not always coupled with more ambitious target setting, and there is a tradeoff between eagerness and feasibility (15).

### 2.3. Research gaps

Several previous studies have provided a basis for understanding the links between quantitative targets and road safety performance, and have established a solid foundation for future target setting practice. However, their findings are largely equivocal, and the association between the characteristics of targets, socio-economic attributes of countries, and their success has not yet been clarified. In particular, the connection between the two core features of a target (i.e., target ambition and target duration) and its achievement remains unclear. Additionally, previous empirical analyses have been performed nationally with small cross-sectional samples, leading to inconsistent results that may not be applicable to other parts of the world.

Therefore, we conducted empirical analysis regionally and globally using panel data representing 20 years of observations from 34 OECD member countries that exhibit comparable attributes in terms of socio-economic development, dedication to achieving road safety targets, implementation of road management systems, and establishment of robust strategy frameworks. These criteria significantly improved the diversity and richness of our samples. Therefore, our findings should be more reliable, applicable, and generalizable.

## 3. Data

### 3.1. Explanatory factors

In addition to the two key attributes of targets, namely target duration and target ambition (with the annual fatality reduction rate as a proxy), other relevant factors that can affect target achievement should also be considered to mitigate model specification issues. Such factors are mainly comprised of safety performance indicators that reflect the road development conditions of an economic entity (37). However, selecting appropriate indicators can be difficult given that potentially correlated criteria do not satisfy the necessary theoretical explanations in the road safety development field and that the quality of research outputs is highly dependent on variable availability and reliability (38). Therefore, the relative factors considered in this study were selected based on an indicator system used in previous studies on road safety development (39, 40), including population density, GDP *per capita*, adult education level, registered vehicles per 1,000 populations, and road network density.

### 3.2. Data summary

**Figure 1:**
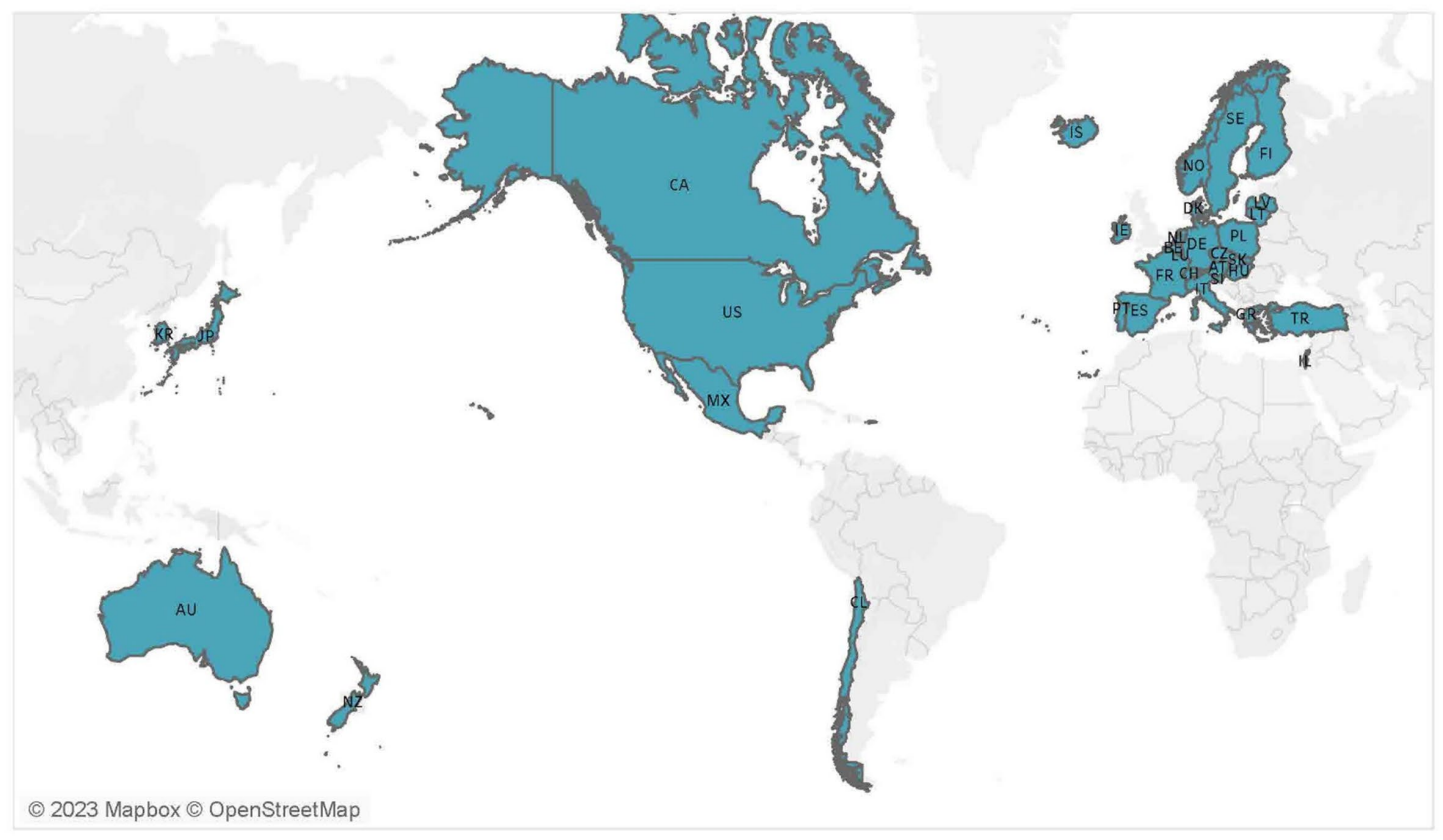
Geographical distribution of the 34 OECD countries examined in this study.

The data on the covariates were collected from various sources for 34 OECD countries.^1^ Figure 1 displays the geographical distribution of the examined countries in this study, and our study investigates within a data set of approximately 20 years (mostly from 1998–2018). Specifically, GDP *per capita*, population density, and adult education level were derived from the World Bank database. Registered vehicles per 1,000 populations and road network density were extracted from OECD statistics and the national statistics of each country. Historical fatality data were obtained from the International Road Traffic and Accident Database 2009–2021 (41). The summary statistics of the datasets considered in this study are presented in Table 1.

**Table 1:** Panel data summary.

Variables		Mean	Std. dev	Min	Max	Observations
Annual reduction rate	Overall	0.057	0.027	0	0.3	*N* = 579
	Between		0.018			*n* = 34
	Within		0.021			T-bar = 17.03
Population density	Overall	194.034	204.006	6.726	963.969	*N* = 579
	Between		203.188			*n* = 34
	Within		9.706			T-bar = 17.03
GDP *per capita*	Overall	33,817.01	20,160.73	2,459.021	123,678.7	*N* = 579
	Between		20,457.11			*n* = 34
	Within		10,909.46			T-bar = 17.03
Adult education level	Overall	29.769	9.687	8.843	51.441	*N* = 474
	Between		9.132			*n* = 32
	Within		4.579			T-bar = 14.81
Registered vehicle per 1,000 populations	Overall	605.275	148.589	208.929	906.502	*N* = 475
	Between		163.793			*n* = 34
	Within		63.768			T-bar = 13.97
Road network density	Overall	132.981	117.885	10.544	510.486	*N* = 424
	Between		112.119			*n* = 34
	Within		9.460			T-bar = 13.25
Target duration	Overall	4.320	2.728	1	13	*N* = 579
	Between		0.969			*n* = 34
	Within		2.576			T-bar = 17.03
Fatality (yearly)	Overall	2,894.417	6,934.374	4	43,510	*N* = 571
	Between		6,736.041			*n* = 34
	Within		1,058.529			T-bar = 16.79

1. The investigation of this study is based on an unbalanced panel in which different cross-sectional individuals (OECD countries in this case study) may contain different number of observations. Due to various reasons within their own jurisdictions, certain countries are unable to provide complete statistical data on traffic safety, resulting in fewer than 20 years of observations for those specific countries. For instance, due to constraints or limitations, the government may lack the capacity to complete statistical data collection for certain years. For some other countries (especially those in the developed world), they may provide fairly complete statistics, some of which might span more than 20 years. Since the number of observed individuals in most countries is around 20, we believe that the time span of our research object is about 20 years (most of them are from 1998–2018). And, the T-bar, therefore, reflects the average number of years of observations across the cross-sectional individuals. The presence of unbalanced panels does not affect the calculation of within-group estimators in the fixed effects (FE) model. For example, FE estimation can be proceeded as usual without significant consequences on the results. Similarly, for the random effects (RE) model, unbalanced panel data does not have a substantial impact either, as long as each individual’s time dimension is considered when conducting feasible generalized least squares (FGLS) estimation through the quasi-demean transformation (42–45). 2. *N* is the total number of observations of the variable across the entire panel. 3. *n* is the number of cross-sectional individuals (number of OECD countries examined in this study).

As shown in Table 1, the average annual reduction rate is 5.57%, which indicates an overview of the approximate annual fatality reduction target for those countries. The annual reduction rate ranges from 0–30%, suggesting different levels of ambition of those countries toward road fatality control. Besides, the average target duration is 4.32 years, which suggests that the quantitative target engagement typically lasts four years or so. As the target duration ranges from 1 year to 13 years, it means that, for all 34 countries, the longest target engagement time reached 13 years, while the shortest was only one year. Different countries have different levels of durability in implementing the targets they have set. Table 1 also presents the overall, between, and within estimation of variable standard deviations (see Appendix A). According to the results, for each variable, it can be observed that the values of the overall, within, and between standard deviations for each variable are considerably large in magnitude relative to their respective means. It means that the data samples utilized in this study are representative of variation, not only from the perspective of the entire panel, but also from the perspective within each cross-sectional individual as well as across cross-sectional individuals.

## 4. Methodology

### 4.1. Target features

#### 4.1.1. Target duration and ambition

Quantitative targets represent the measurable road safety outcomes that a country, jurisdiction, or organization aims to achieve during a given timeframe (11). There are three types of targets: final, intermediate, and institutional. Final targets are considered by all OECD members, while intermediate and institutional targets are employed less commonly. In general, the final target is to reduce the number or percentage of deaths or injuries over a number of years. For example, Argentina set a target in 2016 to reduce the number of deaths caused by road traffic crashes by 30% over ten years (by 2026), and the EU announced a commitment to reduce road deaths by 50% between 2010 and 2020. Two core characteristics can be identified within a target, namely the target duration and ambition.

The government’s ambition and persistence in road fatality control can be discerned from the interplay of two essential elements: the magnitude of quantitative targets they establish as anticipated prerequisites, and the perpetuity and durability of said targets. To explore the impact of the attributes of quantitative road safety targets (i.e., target ambition and duration) on the final target achievement, this study will emphatically explore the influence of two primary explanatory variables, i.e., annual fatality reduction rate and target duration, which are the proxies for, respectively, to level of established quantitative targets and target durability.

In practice, the target duration varies across countries and is typically five or ten years, which typically reflects the timeframe a government sets for the execution and accomplishment of a predetermined quantifiable target. The target ambition can be expressed as the annual reduction rate (*ARR*) of fatalities over the duration. For example, if a country initiates a total reduction rate (*TRR*) over a duration of *n* years, then the *ARR* can be calculated as follows:


$$1-TRR={\left(1-ARR\right)}^{n}$$

meaning


$$ARR=1-{\left(1-TRR\right)}^{\frac{1}{n}}$$

The *TRR*, therefore, is the established quantitative road safety target in road fatality reduction over a period of time, and the *ARR* is the yearly compounded equivalence of *TRR* within the period of time.

#### 4.1.2. Target achievement

The base year is the year before the target year. Based on the actual number of road fatalities in the base year, the number of targeted fatalities at the end of the *n^th^* year of the target duration should be


$$TF_{n}=BF\times \left(1-TRR\right)$$

where *BF* is the number of actual fatalities in the base year and *TF_n_* is the targeted number of fatalities at the end of *n* years.

Because the *ARR* is the annual reduction rate compounded, the equivalent targeted fatalities in the *k^th^* year after the base year within the *n*-year duration (*k* ≤ *n*) should be


$$TF_{k}=BF\times {\left(1-ARR\right)}^{k}$$

where *TF_k_* is the target number of fatalities *k* years after the base year.

If the actual number of fatalities (*AF*) in a year is less than or equal to the targeted number of fatalities *TF_k_*, then the target is achieved. Otherwise, it is not. Therefore, the target completion status (*TCS*) of the *k^th^* year in the duration can be measured as


$$\begin{array}{l} TCS_{k}=\{\begin{array}{l} \mathrm{Yes}\left(1\right),\mathrm{if}\;AF_{k}\leq TF_{k}\\ \mathrm{No}\left(0\right),\mathrm{if}\;AF_{k}>TF_{k} \end{array}\\ k=1,2,\cdots ,n \end{array}$$

To measure the extent to which a target was achieved, the target completion rate (*TCR*) is calculated as follows:


$$\begin{array}{l} TCR_{k}=\frac{TF_{k}}{AF_{k}}\\ k=1,2,\cdots ,n \end{array}$$

If the *TCR* is equal to one, then the target is fully achieved. If the *TCR* is greater than one, then the target is exceeded. If *TCR* is less than one, then the target is not achieved. Clearly, a greater *TCR* indicates greater achievement.

In some cases, a government may define a new target before the original duration expires. This is because governments are sometimes forced to reset targets frequently under external pressure, resulting in relatively short durations of original target execution. In such cases, the newly established target is considered for subsequent calculations and the previous target is forfeited. This concept is referred to as the durability of government goals and represents how long an established target will last. This is reflected by the indicator K, which is the number of years for which a given target has been pursued at a given time point. If a new target is set, then the value of K is reset to one for that year. This indicator can help answer the question of whether a persistent government is more inclined to achieve road fatality reduction targets.

### 4.2. Model specification

#### 4.2.1. Nonlinear logit models for target statuses

Whether a target is successfully achieved is a binary value (i.e., “Yes” or “No”). Therefore, a basic empirical model for target achievement has a nonlinear form. The most widely used regression methodologies for binary classification are the logit and probit models. Given the varying political environments and levels of road development among countries, preferences for target setting and capabilities to control traffic risks also vary. These variations are included in individual effects, but are unobservable in existing databases. For example, local studies on the relationship between target setting and road development performance in different countries typically yield different conclusions [e.g., (12, 14, 15)]. Therefore, we used panel data discrete choice models to account for individual heterogeneity.

Depending on whether an individual effect is correlated with an explanatory variable, heterogeneity can be categorized as fixed or random. A random effect causes an individual effect *u_i_* to exist, but is not correlated with independent variables. For linear panel models, random effect estimators can be obtained using generalized least squares (GLS), whereas, for nonlinear models, GLS is invalid, and maximum likelihood estimation must be employed. Assuming that an individual effect follows a normal distribution $u_{i}∼N\left(0,\sigma_{u}^{2}\right)$ with a probability density function $g\left(u_{i}\right)$ , the conditional probability distribution of individual *i* is


$$\begin{array}{l} y_{\mathrm{it}}=x_{\mathrm{it}}^{'}\beta +u_{i}+\varepsilon_{\mathrm{it}}\\ P\left(y_{\mathrm{it}}=1|x\right)=\Lambda \left(y_{\mathrm{it}}\right)=\Lambda \left(x_{i}^{'}\beta +u_{i}\right)=\frac{e^{x_{i}^{'}\beta +u_{i}}}{1-e^{x_{i}^{'}\beta +u_{i}}} \end{array}$$

This is because we assume that the cumulative probability function for a binary dependent variable follows a logistic distribution. Therefore, the conditional probability density function of *y_it_* can be expressed as


$$\begin{array}{l} f\left(y_{i1},\;y_{i2,}\cdots ,y_{\mathrm{iT}}|x_{\mathrm{it}},\;\beta ,\;u_{i}\right)\\ =\overset{T}{\underset{t=1}{\prod }}{\left[\Lambda \left(u_{i}+x_{\mathrm{it}}^{'}\beta \right)\right]}^{y_{\mathrm{it}}}{\left[1-\Lambda \left(u_{i}+x_{\mathrm{it}}^{'}\beta \right)\right]}^{1-y_{\mathrm{it}}} \end{array}$$

Because the individual effect *u_i_* is unobservable, the joint probability density function of all *y_it_* and *u_i_* should be


$$f\left(y_{i1},\;y_{i2,}\cdots ,\;y_{\mathrm{iT}},\;u_{i}\right)=f\left(y_{i1},\;y_{i2,}\cdots ,\;y_{\mathrm{iT}}|u_{i}\right)\cdot g\left(u_{i}\right)$$

where $g\left(u_{i}\right)$ is the probability density function of $u_{i}∼N\left(0,\sigma_{u}^{2}\right)$ . Therefore, the joint probability density function of all *y_it_* should be

$$\begin{array}{l} l_{i}=f\left(y_{i1},y_{i2,}\cdots ,y_{\mathrm{iT}}\right)\\ =\int_{-\infty }^{+\infty }f\left(y_{i1},y_{i2,}\cdots ,y_{\mathrm{iT}},u_{i}\right)du_{i}\\ =\int_{-\infty }^{+\infty }f\left(y_{i1},y_{i2,}\cdots ,y_{\mathrm{iT}}|u_{i}\right)\cdot g\left(u_{i}\right)du_{i}\\ =\int_{-\infty }^{+\infty }\begin{array}{l} \{\prod_{t=1}^{T}[\Lambda (u_{i}+x_{\mathrm{it}}^{'}\beta )]{}^{y_{\mathrm{it}}}\\ {}[1-\Lambda (u_{i}+x_{\mathrm{it}}^{'}\beta ){]}^{1-y_{\mathrm{it}}}\}\cdot g\left(u_{i}\right)du_{i} \end{array}\\ =\int_{-\infty }^{+\infty }\begin{array}{l} \frac{e^{-u_{i}^{2}/2\sigma_{u}^{2}}}{\sqrt{2\pi }}\{\prod_{t=1}^{T}[\Lambda (u_{i}+x_{\mathrm{it}}^{'}\beta )]{}^{y_{\mathrm{it}}}\\ {}[1-\Lambda (u_{i}+x_{\mathrm{it}}^{'}\beta ){]}^{1-y_{\mathrm{it}}}\}du_{i} \end{array}\\ =\int_{-\infty }^{+\infty }H\left(y_{\mathrm{it}},x_{\mathrm{it}},u_{i}\right)du_{i} \end{array}$$

This is the likelihood function of the panel-based logit model. However, there is no analytical solution to the integral above, but one solution is to apply the “Gauss-Hermite Quadrature” method to obtain a numerical solution (46). The basic formula for this numerical integral can be expressed as


$$\overset{\infty }{\underset{-\infty }{\int }}\upsilon \left(x\right)dx\approx \overset{K}{\underset{k=1}{\sum }}\omega_{k}\cdot e^{\alpha_{k}^{2}}\cdot \upsilon \left(\alpha_{k}\right)$$

where the K is the total quadrature point and the ν(x) is the integrand. Additionally, ω_k_ denotes the weight at each quadrature point and α_k_ denotes the quadrature abscissas. Therefore, if we substitute the individual-level likelihood function into this formula, we obtain


$$l_{i}\approx \sqrt{2}{\hat{\sigma }}_{i}\overset{K}{\underset{k=1}{\sum }}\omega_{k}e^{\alpha_{k}^{2}}H\left(y_{\mathrm{it}},x_{\mathrm{it}},\sqrt{2}{\hat{\sigma }}_{i}\alpha_{k}+{\hat{u}}_{i}\right)$$

The log-likelihood function can be written as


$$\ln\left(l_{i}\right)=\overset{n}{\underset{i=1}{\sum }}\ln\left[\begin{array}{l} \sqrt{2}{\hat{\sigma }}_{i}\omega_{k}e^{\alpha_{k}^{2}}\cdot \frac{e^{\frac{-{\left(\sqrt{2}{\hat{\sigma }}_{i}\alpha_{k}+{\hat{u}}_{i}\right)}^{2}}{2\sigma_{u}^{2}}}}{\sqrt{2\pi }\sigma_{u}}\\ \left\{\begin{array}{l} \overset{T}{\underset{t=1}{\prod }}{\left[\Lambda \left(\sqrt{2}{\hat{\sigma }}_{i}\alpha_{k}+{\hat{u}}_{i}+x_{\mathrm{it}}^{\prime }\beta \right)\right]}^{y_{\mathrm{it}}}\\ {\left[1-\Lambda \left(\sqrt{2}{\hat{\sigma }}_{i}\alpha_{k}+{\hat{u}}_{i}+x_{\mathrm{it}}^{\prime }\beta \right)\right]}^{1-y_{\mathrm{it}}} \end{array}\right\} \end{array}\right]$$

Here, ${\hat{u}}_{i}$ is the estimated individual heterogeneity and ${\hat{\sigma }}_{i}$ is estimated within the standard deviation of individual *i*. Because *u_i_* and *σ_i_* are unobservable, Skrondal and Rabe-Hesketh (47) proposed an adaptive iterative estimation method in which the initial values of ${\hat{\sigma }}_{i}$ and ${\hat{u}}_{i}$ are one and zero, respectively. The estimated ${\hat{\sigma }}_{i}$ and ${\hat{u}}_{i}$ , and likelihood function in the *p*th iteration is

$$\begin{array}{l} l_{i,p}\approx \sum_{k=1}^{K}\sqrt{2}{\hat{\sigma }}_{i,p-1}\omega_{k}e^{\alpha_{k}^{2}}H\left(y_{\mathrm{it}},x_{\mathrm{it}},\Theta_{i,k,p-1}\right)\\ {\hat{u}}_{i,p}=\sum_{k}^{K}\left(\Theta_{i,k,p-1}\right)\frac{\sqrt{2}{\hat{\sigma }}_{i,p-1}\omega_{k}e^{\alpha_{k}^{2}}H\left(y_{\mathrm{it}},x_{\mathrm{it}},\Theta_{i,k,p-1}\right)}{l_{i,p}}\\ {\hat{\sigma }}_{i,p}=\sum_{k}^{K}{\left(\Theta_{i,k,p-1}\right)}^{2}\frac{\sqrt{2}{\hat{\sigma }}_{i,p-1}\omega_{k}e^{\alpha_{k}^{2}}H\left(y_{\mathrm{it}},x_{\mathrm{it}},\Theta_{i,k,p-1}\right)}{l_{i,p}}-{(}^{{\hat{u}}_{i,p})2} \end{array}$$

where $\Theta_{i,k,p-1}$ contains the following information from the last iteration:


$$\Theta_{i,k,p-1}=\sqrt{2}{\hat{\sigma }}_{i,p-1}\alpha_{k}+{\hat{u}}_{i,p-1}$$

Once the iteration converges, we obtain the log-likelihood function and parameter estimation by maximizing likelihood. This process is the adaptive “Gauss-Hermite Quadrature” estimation for random effects in a binary choice.

If the individual effect *u_i_* is correlated with the independent variables, then the panel data estimators should be derived from a fixed-effect model. For a linear model, fixed effect estimators can be determined through a demeaning process within each group to eliminate *u_i_* (48), whereas in a nonlinear panel model, transformation using the demeaning method is infeasible because the relationship between the observed demean $\left(y_{\mathrm{it}}-{\bar{y}}_{\mathrm{it}-1}\right)$ and corresponding demean for latent values $\left(y_{\mathrm{it}}^{*}-{\bar{y}}_{\mathrm{it}-1}^{*}\right)$ is unknown (49). Additionally, another significant issue is “incidental parameters,” where the estimators for fixed effects are inconsistent (50). Such parameters appear because information about *u_i_* can only be derived from a sample with *T* observations for each individual. This insufficient information leads to inconsistent estimations for ${\hat{u}}_{i}$ . It is not only the case that ${\hat{u}}_{i}$ cannot converge to the true value, but that ${\hat{u}}_{i}$ cannot converge at all. The inconsistent estimation of ${\hat{u}}_{i}$ will also lead to the inconsistent estimation of β. To resolve this problem, econometricians typically generate sufficient statistics to implement a conditional maximum likelihood estimation. The definition of a sufficient statistic is that if a statistic Ω contains all the sample information used to estimate the population parameters in the set Ψ, then Ω is a sufficient statistics for Ψ (51). Chamberlain (52) hypothesized that $\theta_{i}=\sum_{t=1}^{T}y_{\mathrm{it}}$ can provide sufficient statistics for *u_i_* because the output *y_it_* is fixed by the individual effect, meaning it can provide all necessary information regarding individual effects. In the simplest case of *T* = 2, there are three possible outcomes for $\theta_{i}=y_{i1}+y_{i2}$ (i.e., zero, one, and two). However, the conditions in which θ_i_ is equal to zero or two provide no information for β estimation. This is because if θ_i_ is equal to zero, then the two y_it_ must both be equal to zero. If θ_i_ is equal to two, then the two y_it_ must both be equal to one. We only need to calculate the conditional probability where θ_i_ is equal to one.


$$\begin{array}{l} P\left(y_{i1}=0,y_{i2}=1|\theta_{i}=1\right)=\frac{P\left(y_{i1}=0,y_{i2}=1\right)}{P\left(y_{i1}=0,y_{i2}=1\right)+P\left(y_{i1}=1,y_{i2}=0\right)}\\ P\left(y_{i1}=1,y_{i2}=0|\theta_{i}=1\right)=\frac{P\left(y_{i1}=1,y_{i2}=0\right)}{P\left(y_{i1}=0,y_{i2}=1\right)+P\left(y_{i1}=1,y_{i2}=0\right)} \end{array}$$

A key assumption is that all y_it_ are independent of each other (53).


$$\begin{array}{l} P\left(y_{i1}=0,y_{i2}=1\right)=\frac{1}{1+e^{x_{i1}^{'}\beta +u_{i}}}\cdot \frac{e^{x_{i2}^{'}\beta +u_{i}}}{1+e^{x_{i2}^{'}\beta +u_{i}}}\\ P\left(y_{i1}=1,y_{i2}=2\right)=\frac{e^{x_{i1}^{'}\beta +u_{i}}}{1+e^{x_{i1}^{'}\beta +u_{i}}}\cdot \frac{1}{1+e^{x_{i2}^{'}\beta +u_{i}}} \end{array}$$

If we substitute these results into the conditional probability calculation, we will obtain


$$\begin{array}{l} P\left(y_{i1}=0,y_{i2}=1|\theta_{i}=1\right)=\frac{e^{x_{i2}^{'}\beta +u_{i}}}{e^{x_{i1}^{'}\beta +u_{i}}+e^{x_{i2}^{'}\beta +u_{i}}}\\ \;=\frac{e^{x_{i2}^{'}\beta_{i}}}{e^{x_{i1}^{'}\beta }+e^{x_{i2}^{'}\beta }}\;\\ \;=\frac{e^{\left(x_{i2}-x_{i1}\right)'\beta }}{1+e^{\left(x_{i2}-x_{i1}\right)'\beta }}\;\\ \;=\Lambda \left[\left(x_{i2}-x_{i1}\right)'\beta \right] \end{array}$$

Similarly,


$$P\left(y_{i1}=0,y_{i2}=1|\theta_{i}=1\right)=\Lambda \left[-\left(x_{i2}-x_{i1}\right)'\beta \right]=1-\Lambda \left[\left(x_{i2}-x_{i1}\right)'\beta \right]$$

exp(*u_i_*) is successfully eliminated based on the mathematical characteristics of the logistic distribution, whereas the probit model could not eliminate this term. The log-likelihood function can then be written as


$$\begin{array}{c} \ln\left(l_{i}\right)=d_{i}\ln\Lambda \left[{\left(x_{i2}-x_{i1}\right)}^{'}\beta \right]\\ +\;\left(1-d_{i}\right)\ln\left\{1-\Lambda \left[{\left(x_{i2}-x_{i1}\right)}^{'}\beta \right]\right\}\cdot 1\left(\theta_{i}=1\right) \end{array}$$

where *d_i_* is a dummy equal to one if (y_i1_ = 0, y_i2_ = 1) and equal to zero if (y_i1_ = 1, y_i2_ = 0). 1(·) is an indicator function suggesting that the information comes from $\theta_{i}=1$ . Maximum likelihood estimation can now be applied.

#### 4.2.2. Linear panel model for target completion rate

To determine how the target annual reduction rate (a reflection of the government’s target ambition) and target duration affect the *TCR*, a linear panel model was adopted.

For fixed effects, a demeaning transformation was performed to eliminate individual heterogeneity.


$$\begin{array}{l} y_{\mathrm{it}}=x_{\mathrm{it}}^{'}\beta +u_{i}+\varepsilon_{\mathrm{it}}\\ {\bar{y}}_{i}={\bar{x}}_{i}^{'}\beta +u_{i}+{\bar{\varepsilon }}_{i}\\ y_{\mathrm{it}}-{\bar{y}}_{i}={\left(x_{\mathrm{it}}-{\bar{x}}_{\mathrm{it}}\right)}^{'}\beta +\left(\varepsilon_{\mathrm{it}}-{\bar{\varepsilon }}_{i}\right) \end{array}$$

Because there are no individual effects in the model following the demeaning transformation, the fixed effect estimator allows the existence of a correlation between *u_i_* and *x_it_*. The fixed effect estimator is consistent if the basic assumptions of a linear estimator, random sampling, and strictly exogenous *x_it_* relative to the idiosyncratic error *ε_it_* are satisfied, even though the conditional mean of *u_i_* is not zero (E(*ε_it_*|*x*) ≠ 0) (43). Then, the coefficients β can be estimated using the GLS as:


$${\hat{\beta }}_{FE-GLS}={\left(\overset{n}{\underset{i=1}{\sum }}x_{i}^{'}M_{T}^{0}x_{i}\right)}^{-1}\left(\overset{n}{\underset{i=1}{\sum }}x_{i}^{'}M_{T}^{0}y_{i}\right)$$

where $M_{T}^{0}$ is the demeaning matrix and the fixed effect estimation can be written as


$$\begin{array}{l} M_{T}^{0}y_{i}=M_{T}^{0}x_{i}+M_{T}^{0}\varepsilon_{i}\\ M_{T}^{0}=I-\frac{1}{T}\left(\begin{array}{cccc} 1 & 1 & \cdots & 1\\ 1 & 1 & \cdots & 1\\ \cdots & \cdots & \cdots & \cdots \\ 1 & 1 & \cdots & 1 \end{array}\right)=\left(\begin{array}{cccc} 1-\frac{1}{T} & -\frac{1}{T} & \cdots & -\frac{1}{T}\\ -\frac{1}{T} & 1-\frac{1}{T} & \cdots & -\frac{1}{T}\\ \cdots & \cdots & \cdots & \cdots \\ -\frac{1}{T} & -\frac{1}{T} & \cdots & 1-\frac{1}{T} \end{array}\right) \end{array}$$

The assumption that the random effect estimators are consistent is stricter than that for the fixed effects. For the random effect estimators, it is required that both the unobserved heterogeneity *u_i_* and idiosyncratic error *ε_it_* cannot be correlated with the explanatory variables (43), meaning


$$\begin{array}{l} \mathrm{cov}\left(x_{\mathrm{it}},u_{i}\right)=0,\;E\left(u_{i}|x_{\mathrm{it}}\right)=0\\ \mathrm{cov}\left(x_{\mathrm{it}},\varepsilon_{\mathrm{it}}\right)=0,\;E\left(\varepsilon_{\mathrm{it}}|x_{\mathrm{it}}\right)=0 \end{array}$$

Random effect estimation follows a quasi-demeaning transformation as


$$\begin{array}{l} y_{\mathrm{it}}=x_{\mathrm{it}}^{'}\beta +u_{i}+\varepsilon_{\mathrm{it}}\\ \theta {\bar{y}}_{i}=\theta {\bar{x}}_{i}^{'}\beta +\theta u_{i}+\theta {\bar{\varepsilon }}_{i}\\ y_{\mathrm{it}}-\theta {\bar{y}}_{i}={\left(x_{\mathrm{it}}-\theta {\bar{x}}_{\mathrm{it}}\right)}^{'}\beta +\left(1-\theta \right)u_{i}+\left(\varepsilon_{\mathrm{it}}-\theta {\bar{\varepsilon }}_{i}\right)\\ \theta =1-\frac{\sigma_{\varepsilon }}{{\left(T\sigma_{u}^{2}+\sigma_{\varepsilon }^{2}\right)}^{1/2}} \end{array}$$

where $\sigma_{u}^{2}$ and $\sigma_{\varepsilon }^{2}$ are the variances of error terms *u_i_* and *ε_it_*, respectively.

It is evident that the individual effect *u_i_* cannot be completely eliminated. If *u_i_* is a fixed effect, but incorrectly incorporated into the random effects estimator, then an endogeneity problem will appear, making the resulting coefficient inconsistent and biased.

The GLS can also be applied to random effect estimators.


$$\begin{array}{l} {\hat{\beta }}_{RE-GLS}={\left(\sum_{i=1}^{n}x_{i}^{'}\Omega^{-1}x_{i}\right)}^{-1}\left(\sum_{i=1}^{n}x_{i}^{'}\Omega^{-1}y_{i}\right)\\ \Omega =\left(\begin{array}{cccc} \sigma_{u}^{2}+\sigma_{\varepsilon }^{2} & \sigma_{u}^{2} & \cdots & \sigma_{u}^{2}\\ \sigma_{u}^{2} & \sigma_{u}^{2}+\sigma_{\varepsilon }^{2} & \cdots & \sigma_{u}^{2}\\ \cdots & \cdots & \cdots & \cdots \\ \sigma_{u}^{2} & \cdots & \sigma_{u}^{2} & \sigma_{u}^{2}+\sigma_{\varepsilon }^{2} \end{array}\right)=\sigma_{u}^{2}l_{T}l_{T}^{'}+\sigma_{\varepsilon }^{2}I_{T} \end{array}$$

Here, Ω is the variance–covariance matrix of the error term, $l_{T}$ is the identity vector, and $I_{T}$ is the identity matrix.

This is because the matrix Ω can also be defined using the demeaning matrix $M_{T}^{0}$ .


$$\begin{array}{l} \Omega =\sigma_{u}^{2}l_{T}l_{T}^{'}+\sigma_{\varepsilon }^{2}I_{T}=\sigma_{\varepsilon }^{2}\left[M_{T}^{0}+\frac{1}{\phi^{2}}\left(I_{T}-M_{T}^{0}\right)\right]\\ \phi =\frac{\sigma_{\varepsilon }}{{\left(T\sigma_{u}^{2}+\sigma_{\varepsilon }^{2}\right)}^{1/2}},\theta =1-\phi \end{array}$$

That is:


$$\begin{array}{l} \Omega^{-1}=\frac{1}{\sigma_{\varepsilon }^{2}}\left[M_{T}^{0}+\phi^{2}\left(I_{T}-M_{T}^{0}\right)\right]\\ \Omega^{-1/2}=\frac{1}{\sigma_{\varepsilon }}\left[M_{T}^{0}+\phi \left(I_{T}-M_{T}^{0}\right)\right] \end{array}$$

and


$$\begin{array}{l} ∵\left[M_{T}^{0}+\phi \left(I_{T}-M_{T}^{0}\right)\right]y_{i}=M_{T}^{0}y_{i}+\phi \left(I_{T}y_{i}-M_{T}^{0}y_{i}\right)\\ \;=\left(y_{i}-{\bar{y}}_{i}\right)+\phi \left[y_{i}-\left(y_{i}-{\bar{y}}_{i}\right)\right]\\ \;=y_{i}-\theta {\bar{y}}_{i} \end{array}$$

$$∴\Omega^{-1/2}y_{i}=\Omega^{-1/2}x_{i}+\Omega^{-1/2}\varepsilon_{i}+\Omega^{-1/2}u_{i}$$

The last equation is the quasi-demeaning model for the random effects estimators. $\sigma_{u}^{2}$ and $\sigma_{\varepsilon }^{2}$ are unobservable population parameters and it is necessary to use sample data to estimate them. ${\hat{\sigma }}_{u}^{2}$ comes from the mean of the sample squared error term in the least squares dummy variable (LSDV) model (see Appendix C.1) and ${\hat{\sigma }}_{\varepsilon }^{2}$ comes from the mean of the sample squared error term of the between-effects (BE) estimators (see Appendix C.2).


$$\begin{array}{l} {\hat{\sigma }}_{u}^{2}=\frac{\overset{N}{\underset{i=1}{\sum }}\overset{T}{\underset{t=1}{\sum }}{\hat{\varepsilon }}_{\mathrm{LSDV},\mathrm{it}}^{2}}{nT-k-1}\\ {\hat{\sigma }}_{\varepsilon }^{2}=\frac{\overset{N}{\underset{i=1}{\sum }}{\hat{e}}_{\mathrm{BE},i}^{2}}{n-k-1} \end{array}$$

Therefore, the estimate from the sample observations is $\hat{\theta }$ , which can be substituted directly into the GLS estimator.


$$\hat{\theta }=1-\frac{{\hat{\sigma }}_{\varepsilon }}{{\left(T{\hat{\sigma }}_{u}^{2}+{\hat{\sigma }}_{\varepsilon }^{2}\right)}^{1/2}}$$

## 5. Results and discussion

### 5.1. Target completion status

Regarding the target completion status, nonlinear logit regression, including Pooled, FE, and RE logit models, was employed to determine its relationship with the annual reduction rate, target duration, and other relevant factors. The basic hypothesis test results for model selection are presented in Table 2 (principles of hypothesis testing, see Appendix B). Table 3 presents the model results for different estimators.

**Table 2:** Hypothesis tests for model selection (Logit).

	Hausman test	LR test for *ρ* = 0
Statistics	χ^2^ = 12.60	χ^2^ = 16.36
Value of *p*	*p* = 0.0498	*p* = 0.0000

**Table 3:** Estimation of different types of logit models (dependent variable: target completion status).

Variables	Models					
	Pooled logit		RE logit		FE logit	
Annual Reduction Rate	−23.501*** (9.156)	z = −2.57 *p* = 0.010	−33.794*** (8.671)	z = −3.90 *p* = 0.000	−40.482*** (9.593)	z = −4.22 *p* = 0.000
Target duration	−0.043 (0.067)	z = −0.64 *p* = 0.523	−0.041 (0.053)	z = −0.76 *p* = 0.448	−0.041 (0.058)	z = −0.69 *p* = 0.487
GDP *per capita*	1.77e-05 (1.09e-05)	z = 1.63 *p* = 0.103	2.52e-05* (1.37e-05)	z = 1.84 *p* = 0.066	6.81e-05*** (2.42e-05)	z = 2.82 *p* = 0.005
Population density	−0.002 (0.001)	z = −1.60 *p* = 0.110	−0.004 (0.002)	z = −1.56 *p* = 0.120	−0.072*** (0.023)	z = −3.18 *p* = 0.001
Adult education level	−0.006 (0.024)	z = −0.26 *p* = 0.795	−0.007 (0.028)	z = −0.26 *p* = 0.798	0.021 (0.058)	z = 0.36 *p* = 0.719
Registered vehicles	−0.003** (0.001)	z = −2.32 *p* = 0.020	−0.004** (0.002)	z = −2.19 *p* = 0.028	−0.007* (0.004)	z = −1.80 *p* = 0.072
Road network density	0.005 (0.003)	z = 1.56 *p* = 0.118	0.008** (0.004)	z = 2.05 *p* = 0.040	0.040** (0.020)	z = 2.00 *p* = 0.045

1. If the value of the estimator is too small, it is denoted using scientific notation. For example, 1.77e−05 = 1.77 × 10^−5^. 2. The standard errors are written in parentheses below the coefficients and hypothesis test results for the significance of individual coefficients are provided. 3. The coefficients are marked with *, **, and ***, if their sample estimators are significant at the 10, 5, and 1% level, respectively. 4. The pooled logit model was estimated using cluster robust standard errors. 5. The RE logit model was estimated using the adaptive “Gauss-Hermite Quadrature” with 16 quadrature points based on the relative distances between coefficients.

As shown in Table 2, the chi-square statistic for the LR test is 16.36 with a value of *p* of zero. This means that the null hypothesis of $\rho =\mathrm{Corr}\left(u_{i}+\varepsilon_{\mathrm{it}},u_{i}+\varepsilon_{im}\right)=0$ is rejected at the 1% significance level, indicating that the variance of individual effects *u_i_* plays an important role in the overall disturbance. Therefore, the pooled logit model is not appropriate in this case. The Hausman statistic value is 12.60, suggesting that the difference between the logit RE and logit FE estimators is systematic at a 5% significance level. The value of *p* indicates that the probability of drawing an incorrect conclusion from the Hausman test is 4.98%. Evidence of no asymptomatic convergence for the logit RE and logit FE estimators implies that the individual effect is correlated with explanatory variables and that logit RE estimators are inconsistent owing to this endogeneity. Therefore, the final model adopted in this study was the FE logit model, where individual fixed effects exist and are believed to be correlated with explanatory variables.

As shown in Table 3, the target annual reduction rate is a significant factor affecting the target status. The coefficients of all three types of logit models are negative and statistically significant at the 1% level. This provides conclusive evidence that the more ambitious the target, the less likely it is to be achieved. In other words, a government that is more ambitious in target setting is generally less effective at target achievement. This result contradicts to relatively earlier studies [e.g., (7, 9, 13)] which indicate that ambitious target setting can be a catalyst for society to take action, but supports some contemporary views of low ambition, which is typically associated with higher rates of achievement. The marginal effect of the FE logit model (see Table 4) reveals that setting a 1% higher annual target reduction rate reduces the likelihood of achieving the target by approximately 0.875%. This probability reduction was approximately 4.859% in the RE logit model estimate. Although the signs of the coefficients for target duration are negative, it cannot be conclusively determined whether a persistent target is less likely to be achieved because the coefficients are not statistically significant. Most other relevant factors are also statistically insignificant, particularly in the pooled logit and RE logit models. Our final adopted model is the FE logit model, while the pooled logit model and RE logit model is ruled out, according to the indication from the hypothesis tests. The FE logit model suggests that population density (1% significance level), GDP *per capita* (1% significance level), registered vehicle numbers (10% significance level) and road network construction (5% significance level) influence target achievement. Their marginal effects suggest that 1 million additional population density per 100 km^2^ will reduce the likelihood of achieving the fatality reduction target by approximately 0.2%, an increase of 1 registered vehicle per 1,000 people in a certain area will reduce the he likelihood of achieving the fatality reduction target by approximately 0.02%, and 1 km of additional road network construction per 100 km^2^ will increase the probability of success by approximately 0.1%. This indicates that it is relatively difficult to implement road safety management in a more densely populated area or in a region with more vehicles owned by citizens, whereas developing a road network system is helpful for preventing traffic fatalities. Besides, it is also evident that quantitative safety targets regarding fatality reduction are more likely to be achieved in a more affluent area (with higher GDP *per capita*).

**Table 4:** Marginal effects of logit models.

Estimators	Pooled logit	RE logit	FE logit
Annual reduction rate	−4.141 (1.500)	−4.859 (1.112)	−0.875 (0.909)
Target duration	−0.008 (0.011)	−0.006 (0.008)	−0.0009 (0.001)
GDP *per capita*	3.12e-06 (1.87e-06)	3.62e-06 (1.87e-06)	1.47e-06 (1.42e-06)
Population density	−0.0004 (0.0002)	−0.0005 (0.0003)	−0.002 (0.002)
Adult education level	−0.001 (0.004)	−0.001 (0.004)	0.0005 (0.001)
Registered vehicles	−0.0006 (0.0002)	−0.0006 (0.0002)	−0.0002 (0.0001)
Road network	0.0009 (0.0005)	0.001 (0.0005)	0.0009 (0.001)

1. If the value the estimator is too small, it is denoted using scientific notation. For example, 2. The standard errors are written in parentheses below the marginal effects. 3.12e−06 = 3.12 × 10^−6^.

### 5.2. Target completion rate

A linear regression model was applied to determine the factor contributions to the *TCR*. The results of the hypothesis tests for the final model selection are presented in Table 5 (principles of hypothesis testing, see Appendix B). Table 6 presents the results for different types of estimators.

**Table 5:** Hypothesis tests for model selection (Linear).

Type of hypothesis testing	Statistics	Value of *p*
Hausman test	χ^2^ = 9.40	*p* = 0.1523
Bootstrap Hausman test	χ^2^ = 23.88	*p* = 0.0012
Overidentification test of RE over FE	χ^2^ = 21.154	*p* = 0.0035
BP LM test for random effect	χ^2^ = 57.22	*p* = 0.0000
Between heteroskedasticity test	χ^2^ = 10922.02	*p* = 0.0000
White test for heteroskedasticity in Pooled OLS	χ^2^ = 106.44	*p* = 0.0000

**Table 6:** Estimation results from different types of linear models (dependent variable: target completion rate).

Estimators	Pooled OLS		RE		FE	
Annual reduction rate	−1.875*** (0.598)	*t* = −3.13 *p* = 0.004	−2.175*** (0.757)	z = −2.87 *p* = 0.004	−2.386*** (0.863)	*t* = −2.76 *p* = 0.010
Target duration	−0.005 (0.006)	*t* = −0.92 *p* = 0.363	−0.004 (0.006)	z = −0.65 *p* = 0.513	−0.004 (0.006)	*t* = −0.68 *p* = 0.504
GDP *per capita*	−6.41e-09 (1.15e-06)	*t* = −0.01 *p* = 0.996	1.03e-06 (1.07e-06)	z = 0.96 *p* = 0.337	3.35e-06*** (1.22e-06)	*t* = 2.75 *p* = 0.010
Population density	−0.00007 (0.00008)	*t* = −0.87 *p* = 0.393	−0.0001 (0.0001)	z = −1.16 *p* = 0.245	−0.003*** (0.0008)	*t* = −3.40 *p* = 0.002
Adult education level	−0.0005 (0.0016)	*t* = −0.32 *p* = 0.751	−0.0005 (0.0016)	z = −0.30 *p* = 0.762	0.0003 (0.003)	*t* = 0.1 *p* = 0.92
Registered vehicles	−0.00003 (0. 0001)	*t* = −0.30 *p* = 0.766	−0.00008 (0.00011)	z = −0.79 *p* = 0.432	−0.0002 (0.0002)	*t* = −1.27 *p* = 0.215
Road network	0.0003* (0.0002)	*t* = 1.77 *p* = 0.087	0.0005** (0.0002)	z = 1.96 *p* = 0.050	0.002*** (0.0006)	*t* = 2.80 *p* = 0.009

1. If the value of an estimator is too small, then it is denoted using scientific notation. For example, −6.14e−09 = −6.14 × 10^−9^. 2. The standard errors are written in the parentheses below the coefficients and hypothesis test results for the significance of individual coefficients are provided. 3. The coefficients are marked with *, **, and *** if their sample estimates are significant at the 10, 5, and 1% level, respectively. 4. All models were estimated with clustered robust standard errors.

According to the results of the Breuch-Pagan LM test for random effects in Table 5, the null hypothesis of no variance in individual effects ( $\sigma_{u}^{2}=0$ ) is rejected, suggesting that the pooled OLS can be ruled out based on individual heterogeneity. This also indicates that a panel data regression model that can account for the heterogeneity of each cross-sectional individual is more reasonable, whereas a direct cross-sectional data pooled OLS will essentially trigger heterogeneity bias. The traditional Hausman test suggests that the differences between the FE and RE linear estimators are not systematic. However, this test result is invalid based on the conditional heteroscedasticity of the residuals. The White test conducted after the pooled OLS suggests that conditional heteroskedasticity exists, even though the observations are not clustered by country. Additionally, by conducting a Wald test for between-group heteroskedasticity (54), we rejected the null hypothesis of identical between-group variance of individuals. In other words, between-group heteroskedasticity exists and the clustering of robust standard errors is essential. Unlike the traditional Hausman test, the bootstrapping Hausman test suggests that the RE estimators cannot converge to the FE estimators. Therefore, u_i_ is not a random effect, but a fixed effect. Furthermore, the over-identification test results indicate that the RE estimate cannot provide any additional information regarding the lack of correlation between *u_i_* and *x_it_*. Therefore, the traditional Hausman test results are unreliable and the FE estimator is more appropriate.

Table 6 reveals that an increasing target annual reduction rate in all model types significantly reduces the target completion rate. The FE estimators indicate that targeting an annual reduction rate of 1% will reduce the achievement rate of road fatality control by 2.386%. This finding agrees well with the conclusions drawn from a previous logit model. A more ambitious target will not only reduce the likelihood of achievement, but also the target achievement rate. However, the target duration does not significantly influence the target completion rate. This is likely because it does not matter whether a government frequently changes its quantitative target setting, but the prerequisite for achievement is realistic goals in combination with pragmatic actions. Other factors are not statistically significant. Only the population density, *per capita* income level and road network can be considered to make any contribution to the target completion rate in the FE model estimates, but their marginal contributions are relatively small. This conclusion is also consistent with the previous results obtained through FE logit estimation. A larger road network system not only increases the likelihood of target achievement, but also helps societies control traffic risk, whereas population density has the opposite effect. Similarly, higher income areas have higher target completion rate.

## 6. Conclusion and implications

### 6.1. Concluding remarks and policy implications

In this study, we employed nonlinear and linear panel models to explore the links between target characteristics (i.e., target duration and ambition) and achievement (i.e., target completion status and rate) using panel data representing 20 years of observations from 34 OECD member countries. The results indicate that a more ambitious quantitative target setting is a viable strategy for achieving road fatality reduction goals, but that more ambitious targets for expected fatalities reduce both the likelihood and extent of target achievement. A more ambitious quantitative target setting typically indicates a more ambitious government in terms of traffic fatality control. In contrast to some early studies indicating that ambitious target setting can motivate society to take action, our results suggest that overly ambitious targets may lead to demotivation. This is because the main driver for achieving goals is not the ambition of plans made by policymakers, but the pragmatic actions that are actually taken based on realistic target proposals (55, 56). This implication supports modern views regarding road development that there should be a tradeoff between the quantitative target level and target achievement capability of society, and that excessive ambition may lead to reduced accomplishment.

Duration was determined to be an insignificant factor for the target achievement status and rate. In other words, whether a government frequently makes adjustments and sets a new target without the original target ending will not significantly affect target achievement. Other relevant variables, including population density, road network systems, adult education level, and registered vehicles contribute very little to the target achievement status and rate. Most results were statistically insignificant and two factors related to reginal road development level, namely population density and road network size, fundamentally affected target achievement, but their marginal effects were relatively small. Fatality reduction targets are more likely to be achieved in a more affluent area with higher GDP *per capita*. Vehicle ownership in the region only affects the likelihood of achieving the target, but not the target completion rate.

The main implication of this study is that governments should focus on pragmatic countermeasures instead of presenting lofty ambitions to the public without sufficient executive power. Realistic road safety development plans account for designing a cost-effective strategy because a high-level target is typically costly, which can be discouraging to the public. Therefore, it will be worthwhile to investigate feasible cost–benefit strategies for road safety development and measure how they can promote the likelihood of injury prevention in future studies. Practical target planning requires the government to consider local macroeconomic factors and existing traffic construction levels, rather than annual traffic fatality statistics.

Although previous studies have laid the groundwork for comprehending the relationship between quantitative targets and road safety performance, their findings are equivocal; our study aims to address this ambiguity. Table 7 summarizes studies exploring the association between quantitative safety targets and injury/fatality prevention. Discrepancies in the research findings can be attributed to the following factors.

1. Various studies have explored different countries/regions as their study objects, resulting in divergent research findings and outcomes (i.e., individual heterogeneity caused these disparities). Considering that different countries/regions, as study objects, may embrace different individual characteristics that would impact road safety performance (e.g., spatial idiosyncrasy and different social development levels), disparate research findings would occur when selecting different study objects. Therefore, using panel data empirical methodologies in our study helps mitigate the influence of individual heterogeneity, as individual heterogeneity is eliminated in these models through numerical transformation. Additionally, taking all OECD countries as the study objects would bring research findings that can provide basic guidance and reference for practitioners worldwide, as OECD members could be representative of individuals with different socioeconomic development levels.
2. Another primary factor explaining the discrepancies in the results among previous studies is the different investigation periods employed. As we have summarized in Table 7, earlier studies suggest that more ambitious targets will bring about more effective injury/fatality prevention, whereas contemporary studies indicate that more ambitious targets do not necessarily result in more effective injury/fatality prevention and, in some cases, may even have a negative association with such outcomes. Our study incorporated a more recent data set with 20 years of longitudinal observations, and our research findings are in accordance with those of contemporary studies. This is because in the early years, when the concept of identifying quantitative targets in road safety risk management had just emerged, many countries were in the initial stage of adopting quantitative safety targets in governance plans. Consequently, their attempts and applications are not yet fully developed or mature, and any proactive measures are likely to yield positive results. At this stage, more ambition in target setting could be highly motivating. However, as the concept, implementation criteria, and utility of using quantitative safety targets in road safety risk management have become clearer in many countries, the adoption of this approach has become widespread for a considerable time, and adjusting the level of ambition in setting quantitative targets makes it difficult to achieve the same positive effects as in earlier periods. As the use of quantitative safety targets in road safety risk management has been normalized and widely adopted by various countries, the ambition itself to set quantitative safety targets will no longer have a direct incentive effect, whereas an excessively ambitious target will be demotivating because having a desire without possessing high-level execution ability will result in incomplete implementation. Therefore, an extrapolation is that the prerequisite to improve the effectiveness of the quantitative road safety management mode does not stop at how ambitious it should be, but more attention should be paid to other levels, including implementation, supervision, feasibility, and cost control.
3. An exception in contemporary studies is the research conducted in Cambodia in 2017 (12), which found that an ambitious target set was expected and forecasted to effectively reduce traffic fatalities in the future. This exception can be attributed to the individual heterogeneity (i.e., idiosyncratic characteristics) of Cambodia. Given that Cambodia is a low-income country in the initial stage of safety target management, its confounding factors such as the presence of detailed road safety programs, long-term commitments, and expenditures are peculiar (12). In this context, ambitions regarding safety targets are likely to provide strong motivation. Moreover, although most previous studies have not explored the effect of target duration, our finding that target duration is insignificantly related to final target achievement aligns with a contemporary study conducted in 2014 (8).

**Table 7:** Typical research exploring the association between quantitative safety targets and road injury/fatality prevention.

Literature	Period	Country/Region	Ambition	Duration
Elvik (7)	1980s	Norwegian counties	+	Ø
Elvik (33)	1970–1998	14 OECD developed countries	+	Ø
Wong et al. (13)	1981–1999	14 OECD developed countries	+	Ø
Wong and Sze (9)	1970–2000	30 OECD members	+	Ø
Belin et al. (14)	1972–2007	Sweden	–	Ø
Allsop et al. (6)	1970–1998	14 OECD developed countries	+	Ø
Gargett et al. (15)	1971–2010	Australia	–	Ø
Shen et al. (34)	Until 2008	27 EU countries	–	Ø
Sze et al. (8)	1981–2009	29 OECD members	–	×
Commandeur et al. (12)	1995–2020 (with forecasting)	Cambodia	+	Ø
This study	1998–2018	34 OECD members	–	**×**

1. Sign “+” refers to the positive association, which means that this study finds that higher target ambition or longer duration will bring better injury/fatality prevention outcomes. 2. Sign “–” indicates that the study finds that higher target ambition or longer duration does not necessarily mean better injury/fatality prevention outcomes, or a negative relationship between target setting ambition/duration and injury/fatality prevention outcomes. 3. Sign “Ø” means that the association is not explored in the study. 4. Sign “×” suggests that the study finds the association is not significant.

### 6.2. Limitations and further studies

There are a few promising extensions to this study based on several limitations. First, our analysis was performed based on econometric models using empirical data. The relationships between quantitative target setting factors remain a hotly debated topic in academia. Therefore, it is important to develop a theoretical knowledge base that fits the empirical results of this study. Second, the empirical models used in this study may suffer from the issue of bidirectional causality, where a government is more inclined to define an ambitious target to force society to take action based on a large number of reported fatalities [e.g., (57, 58)]. This could be explained by multiple equations in panel data models. To resolve this issue, highly developed theoretical knowledge regarding road safety development is required to identify appropriate variables for constructing panel-formed systematic equations. This is a promising direction for future research. Finally, more socio-economic factors could be considered covariates. Future advances from these points suggest a more in-depth longitudinal analysis with more potential variables under a larger panel data sample.

Formulating quantitative road safety targets through governance programs, particularly those equipped with standardized frameworks, has been a fledging concept within the past 20–30 years, receiving more and more attention from the international community and people. Some authoritative international organizations, such as the European Commission and the United Nations, have established unified norms and standards for setting road safety targets, and the management of quantitative safety targets by countries worldwide operates within those fundamental frameworks (11, 59). Setting quantitative targets as practical task objectives to guide implementation has become a customary practice of road safety governance in various countries. Some public evidence can be found to indicate that concrete actions and measurements have been taken in response to the quantitative safety targets that represent the national strategies. For example, the insights of Vision Zero in road fatality control have been adopted by numerous countries and regions all over the world, and their strategic plans, execution, and effects are well documented (4). For OECD countries, road safety targets are also widely adopted to their traffic risk control. There is also evidence suggesting that the implementation of these measures has been monitored and reported, and outcomes of their efforts have been recorded [e.g., (60–62)]. In terms of this evidence, our study can objectively reflect the impact of quantitative safety targets’ ambition on their target achievement to a certain extent, given the perspective of implementation and supervision publicly reflected. Undeniably, the effective management of road safety risks hinges not only on safety target, but also on the meticulous implementation measures and diligent supervision in place. Therefore, future extensions related to implementation and supervision levels should be considered. Future directions could be posted on how the attributes of safety target would affect the effectiveness of execution and supervision, and how the implementation of monitoring, in turn, affect the level of target setting and final achievement. By that time, a complete linkage of the whole steps for road safety risk management (adaption, implementation, following-up, monitoring, evaluation) would be established, and the mechanism it reflects will be a comprehensive guide to road safety development.

## Data availability statement

The raw data supporting the conclusions of this article will be made available by the authors, without undue reservation.

## Author contributions

HC: Data curation, Formal analysis, Methodology, Writing – original draft. YG: Data curation, Formal analysis, Methodology, Writing – original draft. YL: Data curation, Formal analysis, Methodology, Writing – original draft. JZ: Data curation, Formal analysis, Methodology, Writing – original draft. YW: Data curation, Formal analysis, Methodology, Writing – original draft. LY: Data curation, Investigation, Validation, Writing – original draft. JH: Data curation, Writing – original draft, Investigation, Validation. HW: Data curation, Investigation, Validation, Writing – original draft. YB: Data curation, Investigation, Validation, Writing – original draft. YM: Data curation, Investigation, Validation, Writing – original draft. FC: Conceptualization, Project administration, Supervision, Writing – review & editing.

## Footnotes

^1^OECD currently counts 38 member countries. Colombia (CO), Costa Rica (CR), Estonia (EE), and United Kingdom (UK) were not included in this study due to data unavailable.
