# Supplementary material for: Impact of quantitative safety targets on road fatality reduction: an empirical support toward governance plan
Source: Front Public Health. 2023 Nov 7;11:1271328. doi: 10.3389/fpubh.2023.1271328 (PMC10662322; doi:10.3389/fpubh.2023.1271328)
Supplement: Supplementary file 1 [file Data_Sheet_1.docx]

**Appendix A**

When describing variables from a panel data set, there are three types of standard deviations that are commonly considered: overall, between, and within.

**Overall standard deviation**

Overall standard deviation measures the variability of the variables across all cross-sectional individuals and time periods in the panel data set. It provides an overall measurement of dispersion and variations for the entire dataset. For a certain variable *x_it_* (*i* refers to the cross-sectional individuals, and *t* refers to the time series observations), the overall standard deviation is as follow:

$$S^{2}=\frac{1}{N}\sum_{i=1}^{n} \sum_{t=1}^{T_{i}} (x_{it}-\bar{x})^{2}$$

*T_i_* is the number of observations within the cross-sectional individual *i*, and *n* is the number of cross-sectional individuals. N is the total number of observations for the entire data set, across all cross-sectional individuals and time periods.

$$N=\sum_{i=1}^{T_{i}} nt_{i}$$

The overall variation is defined upon the sum of squared deviations from the overall mean $\bar{x}$, which is the arithmetic mean for the entire data set, across all cross-sectional individuals and time periods:

$$\bar{x}=\frac{1}{N}\sum_{i=1}^{n} \sum_{t=1}^{T_{i}} x_{it}$$

**Between standard deviation**

Between standard deviation examines the variability of the variables across cross-sectional individuals at specific points in time. It indicates the differences or heterogeneity among cross-sectional individuals when hold the time fixed. For a certain variable *x_it_*, the between standard deviation is as follows:

$$S_{b}^{2}=\frac{1}{n}\sum_{i=1}^{n} (\bar{x}_{i}-\bar{x})^{2}$$

**Within standard deviation**

Within standard deviation captures the variability of the variables within each cross-sectional individual (aka cross-sectional variation) over time. It helps assess the extent of variation or volatility observed within each cross-sectional individual across different time periods. For a certain variable *x_it_*, the within standard deviation is as follows:

$$S_{w}^{2}=\frac{1}{N}\sum_{i=1}^{n} \sum_{t=1}^{T_{i}} (x_{it}-\bar{x}_{i})^{2}$$

The within standard deviation is defined upon the sum of squared deviations from the individual mean $\bar{x}$*_i_*, which is the arithmetic mean within each cross-sectional individual:

$$\bar{x}_{i}=\frac{1}{T_{i}}\sum_{t=1}^{T_{i}} x_{it}$$

**Appendix B**

The pooled OLS or logit model was ruled out because the existence of heterogeneity leads to biased results. A hypothesis test was conducted to verify the existence of individual effects. The null hypothesis was that the population variance of *u_i_* is zero based on sample information [[63](#_ENREF_63), [64](#_ENREF_64)].

$$\begin{matrix} H_{0}\text{：} \sigma_{u}^{2}=0 \\ H_{1}\text{：} \sigma_{u}^{2}>0 \end{matrix}$$

$$LM=\frac{nT}{2(T-1)}\left[\frac{\sum_{i=1}^{n} \left(\sum_{t=1}^{T} e_{it}\right)^{2}-1}{\sum_{i=1}^{n} \sum_{t=1}^{T} e_{it}^{2}}\right]\sim\chi_{1}^{2}$$

Here, *e_it_* is the error term of a direct pooled OLS regression

$$y_{it}=x_{it}^{'}\beta+e_{it}$$

The LM statistics followed a chi-square distribution with one degree of freedom. If the LM statistic is greater than the critical values of the chi-squared quantiles at a given significance level or if the corresponding p-value is less than the significance threshold, then the null hypothesis is rejected and the pooled estimators are invalid based on heterogeneity.

Another verification method is to determine the autocorrelation coefficient of the hybrid error term (*u_i_* +*ε_it_*) [[65](#_ENREF_65)]. The covariance and correlation coefficients among the error terms are

$$\mathrm{cov}(u_{i}+\varepsilon_{it},u_{i}+\varepsilon_{im})=\left\{\begin{matrix} \sigma_{u}^{2} \\ \sigma_{u}^{2}+\sigma_{\varepsilon}^{2} \end{matrix}\right.\begin{matrix} t\neq m \\ t=m \end{matrix}$$

$$\therefore\rho=Corr(u_{i}+\varepsilon_{it},u_{i}+\varepsilon_{im})=\frac{\sigma_{u}^{2}}{\sigma_{u}^{2}+\sigma_{\varepsilon}^{2}}$$

The greater the value of ρ, the more important the individual effects in the hybrid error term, and the more appropriate it is to use FE or RE models instead of pooled estimators.

For both linear and nonlinear cases, it is important to select between FE and RE estimators. The most widely used methodology for making this choice in panel data econometrics is the Hausman Test, where the RE estimator of a large sample converges to the FE estimator if *u_i_* is not correlated with *x_it_* [[66](#_ENREF_66)]. Therefore, this hypothesis test can be written as

$$\begin{matrix} H_{0}\text{：} E(u_{i}|x)=0,RE estimators converge to FE estimators \\ H_{1}\text{：} E(u_{i}|x)\neq0,Difference between RE and FE estimators are systematic \end{matrix}$$

$$W=(\hat{\beta}_{FE}-\hat{\beta}_{RE})'[\hat{V}ar(\hat{\beta}_{FE})-\hat{V}ar(\hat{\beta}_{RE})]^{-1}(\hat{\beta}_{FE}-\hat{\beta}_{RE})\sim\chi_{k}^{2}$$

The Hausman statistic W is asymptotic to a chi-square distribution with *k* degrees of freedom, where *k* is the dimension of the estimators. If the W statistic is significant at a certain confidence level, then the null hypothesis is rejected, indicating that the difference between the FE and RE estimators is systematic. In this case, the individual effect is a fixed effect that is correlated with independent variable coefficients. Mistakenly utilizing the RE estimator results in an endogeneity problem and yields biased and inconsistent estimates. Otherwise, when the W statistic is not significant, the RE estimators are selected for their efficiency. The Hausman test can be applied to both the linear and logit panel models used in this study. However, the traditional Hausman test is invalid if the error term is heteroscedastic and a bootstrapping Hausman test is used with clustered robust standard errors [[67](#_ENREF_67)].

Another solution (for linear panel models only) applies the following assisted regression[[1]](#footnote-1), where a robust standard error can be used to test whether an individual effect is random [[45](#_ENREF_45)].

$$y_{it}-\hat{\theta}\bar{y}_{i}=(x_{it}-\hat{\theta}\bar{x}_{i})'\beta+(x_{it}-\bar{x}_{i})'\eta+[(1-\hat{\theta})u_{i}+(\varepsilon_{it}-\hat{\theta}\bar{\varepsilon}_{i})]$$

$$\begin{matrix} H_{0}\text{：} \eta=0 \\ H_{1}\text{：} \eta\neq0 \end{matrix}$$

This test can be performed in cases with heteroskedasticity. When η = 0, this equation is equivalent to the linear RE model. Therefore, we can substitute the $\hat{\theta}$ value from robust RE estimation into this equation. If *u_i_* is a random effect, then OLS estimation for this assisted regression is consistent and $\mathop{p\lim}\limits_{n\text{→}\infty} \hat{\eta}=\eta=0$. Otherwise, the OLS estimation for this regression is inconsistent based on the correlation between $[(1-\hat{\theta})u_{i}+(\varepsilon_{it}-\hat{\theta}\bar{\varepsilon}_{i})]$ and $(x_{it}-\bar{x}_{i})'$. In this case, $\mathop{p\lim}\limits_{n\text{→}\infty} \hat{\eta}=\eta^{*}\neq\eta=0$, so the test is equivalent to a single-parameter hypothesis test for whether η = 0. The main principle is that if the null hypothesis is rejected, then the random effect is ruled out.

Finally, the prerequisite for both FE and RE estimators to be consistent is that the idiosyncratic error ε_it_ cannot be correlated with the independent variables. We are concerned with how the selected factors affect the target achievement rate in general for all OECD members, rather than the marginal benefit for each country.

**Appendix C**

**LSDV model**

The LSDV model can be estimated by including a set of dummy variables (one for each cross-sectional unit or each individual country across the 34 OECD members) to represent the individual effect [[43](#_ENREF_43)].

$$d_{1,it}=\left\{\begin{matrix} 1, i=1 \\ 0, i\neq1 \end{matrix}\right., d_{2,it}=\left\{\begin{matrix} 1, i=2 \\ 0, i\neq2 \end{matrix}\right., \cdots, d_{n,it}=\left\{\begin{matrix} 1, i=n \\ 0, i\neq n \end{matrix}\right.$$

The LSDV model adds dummies representing each individual to the panel data regression model.

$$y_{it}=x_{it}^{'}\beta+\delta_{2}d_{2,it}+\delta_{3}d_{3,it}+\cdots+\delta_{n}d_{n,it}+\varepsilon_{LSDV,it}$$

To avoid perfect collinearity, we included only (*n*−1) dummies. Here, ε_LSDV,it_ is the error term of the LSDV model. By directly applying the OLS method to the LSDV model, this error term can be estimated using regression residuals, which are also used to calculate the variance estimator of the individual heterogeneity error term in an RE panel data regression model.

$$\hat{\sigma}_{u}^{2}=\frac{\sum_{i=1}^{N} \sum_{t=1}^{T} \hat{\varepsilon}_{LSDV,it}^{2}}{nT-k-1}$$

**Between-effects estimator**

Another estimator for panel data is the between-effects (BE) estimator, which ignores the variation within each individual and considers variations between cross-sectional individuals [[43](#_ENREF_43)]. The BE estimator is based on the within-group transformation equation.

$$\begin{matrix} y_{it}=x_{it}^{'}\beta+u_{i}+\varepsilon_{it} \\ \bar{y}_{i}=\bar{x}_{i}^{'}\beta+u_{i}+\bar{\varepsilon}_{i} \\ =\bar{x}_{i}^{'}\beta+u_{BE,i}+\bar{\varepsilon}_{BE,i} \\ =\bar{x}_{i}^{'}\beta+e_{BE,i} \end{matrix}$$

The second equation above defines the regression of the individual-specific sample means of the explained variable on the individual-specific means of the explanatory variables using GLS in a cross-sectional regression of n observations. The BE estimator is expressed as

$$\begin{matrix} \hat{\beta}_{BE-GLS}=(\frac{1}{T^{2}}\sum_{i=1}^{n} x_{i}^{'}l_{T}l_{T}^{'}x_{i})^{-1}(\frac{1}{T^{2}}\sum_{i=1}^{n} x_{i}^{'}l_{T}l_{T}^{'}y_{i}) \\ =(\sum_{i=1}^{n} x_{i}^{'}l_{T}l_{T}^{'}x_{i})^{-1}(\sum_{i=1}^{n} x_{i}^{'}l_{T}l_{T}^{'}y_{i}) \end{matrix}$$

The BE regression residuals are estimated based on this estimator and used to derive the variance estimator of the idiosyncratic error term in an RE panel data regression model.

$$\hat{\sigma}_{\varepsilon}^{2}=\frac{\sum_{i=1}^{N} \hat{e}_{BE,i}^{2}}{n-k-1}$$

**References**

43. Biorn, E., *Econometrics of Panel Data: Methods and Applications*. 2016, Oxford: Oxford University Press.

45. Wooldridge, J.M., *Econometric analysis of cross section and panel data: 2. ed*. 2 ed. Economics. Vol. 1. 2010, Cambridge, Mass. u.a: The MIT Pr.

63. Baltagi, B.H. and Q. Li, *A lagrange multiplier test for the error components model with incomplete panels.* Econometric Reviews, 1990. **9**(1): p. 103-107.

64. Breusch, T.S. and A.R. Pagan, *The Lagrange Multiplier Test and its Applications to Model Specification in Econometrics.* The Review of economic studies, 1980. **47**(1): p. 239-253.

65. Arellano, M., *Panel data econometrics*. Advanced texts in econometrics. 2003, Oxford: Oxford University Press.

66. Hausman, J.A., *Specification Tests in Econometrics.* Econometrica, 1978. **46**(6): p. 1251-1271.

67. Kapetanios, G., *TESTING FOR EXOGENEITY IN THRESHOLD MODELS.* Econometric theory, 2010. **26**(1): p. 231-259.

1. Because the panel dataset used in this study is unbalanced, the over-identification test for the RE model over the FE model was performed, which is equivalent to this assistant regression test. This test is only applicable to linear panel data models. [↑](#footnote-ref-1)
